# Supplementary figures and images for: Do psychiatric diseases follow annual cyclic seasonality?
Source: PLoS Biol. 2021 Jul 19;19(7):e3001347. doi: 10.1371/journal.pbio.3001347 (PMC8345894; doi:10.1371/journal.pbio.3001347)

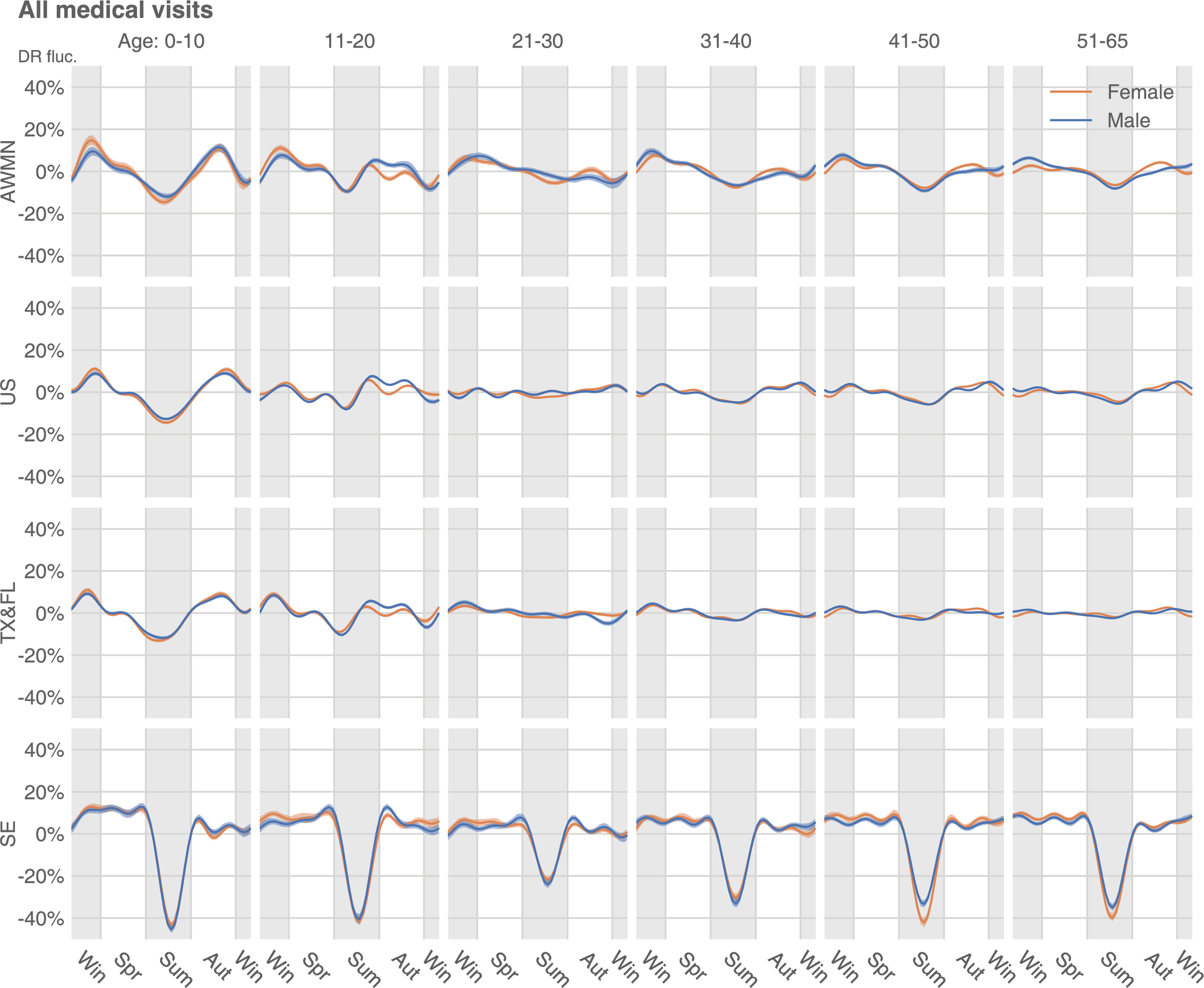

Supplement: S1 Fig — The data underlying this figure can be found in https://doi.org/10.5061/dryad.vdncjsxv6. AK, Alaska; AWMN, xxx; FL, Florida; MT, Montana; ND, North Dakota; SE, Sweden; TX, Texas; WA, Washington. (TIF) [file pbio.3001347.s011.tif]

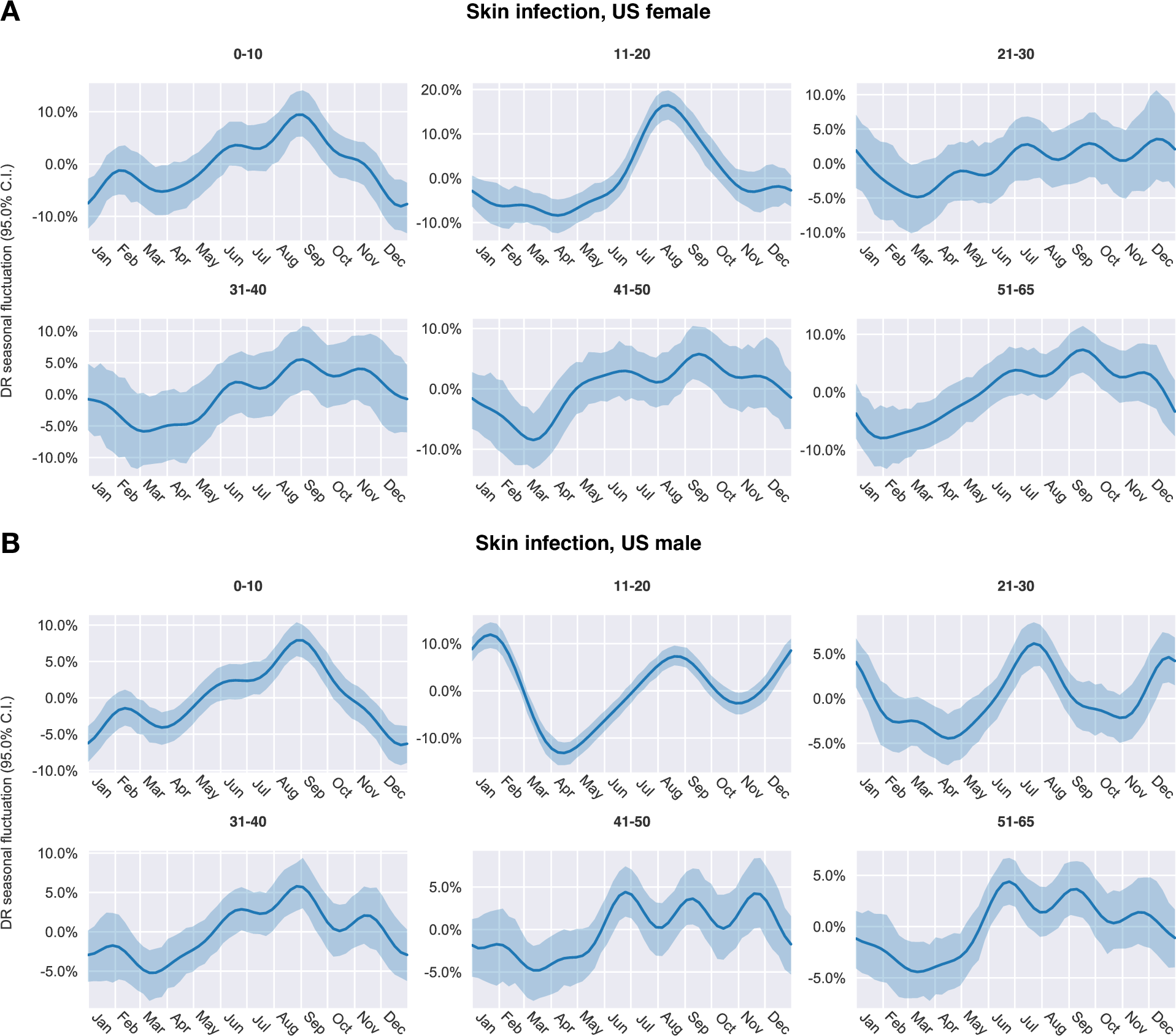

Supplement: S2 Fig — The data underlying this figure can be found in https://doi.org/10.5061/dryad.vdncjsxv6. (TIF) [file pbio.3001347.s012.tif]

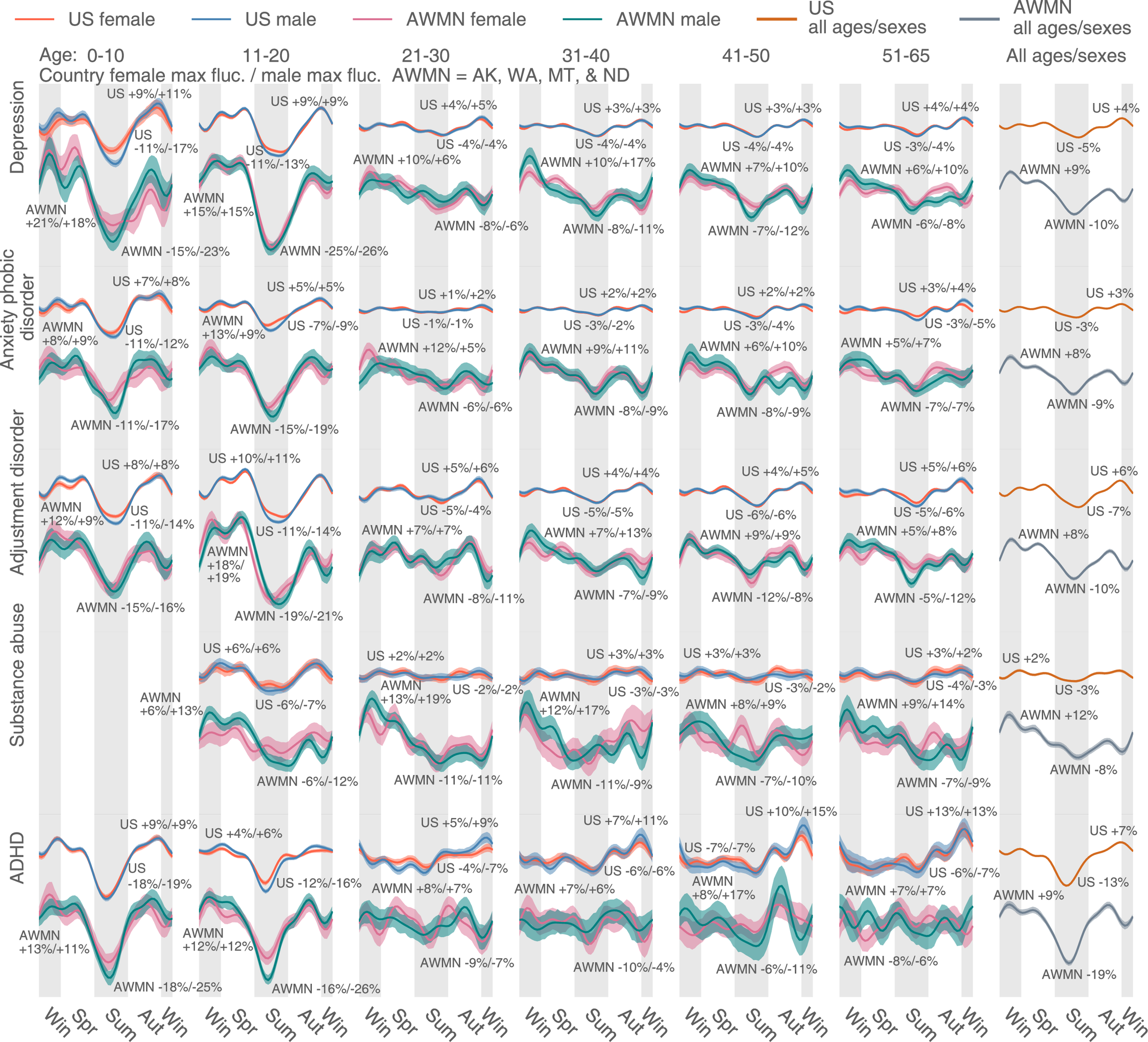

Supplement: S3 Fig — The data underlying this figure can be found in https://doi.org/10.5061/dryad.vdncjsxv6. AK, Alaska; MT, Montana; ND, North Dakota; WA, Washington. (TIF) [file pbio.3001347.s013.tif]

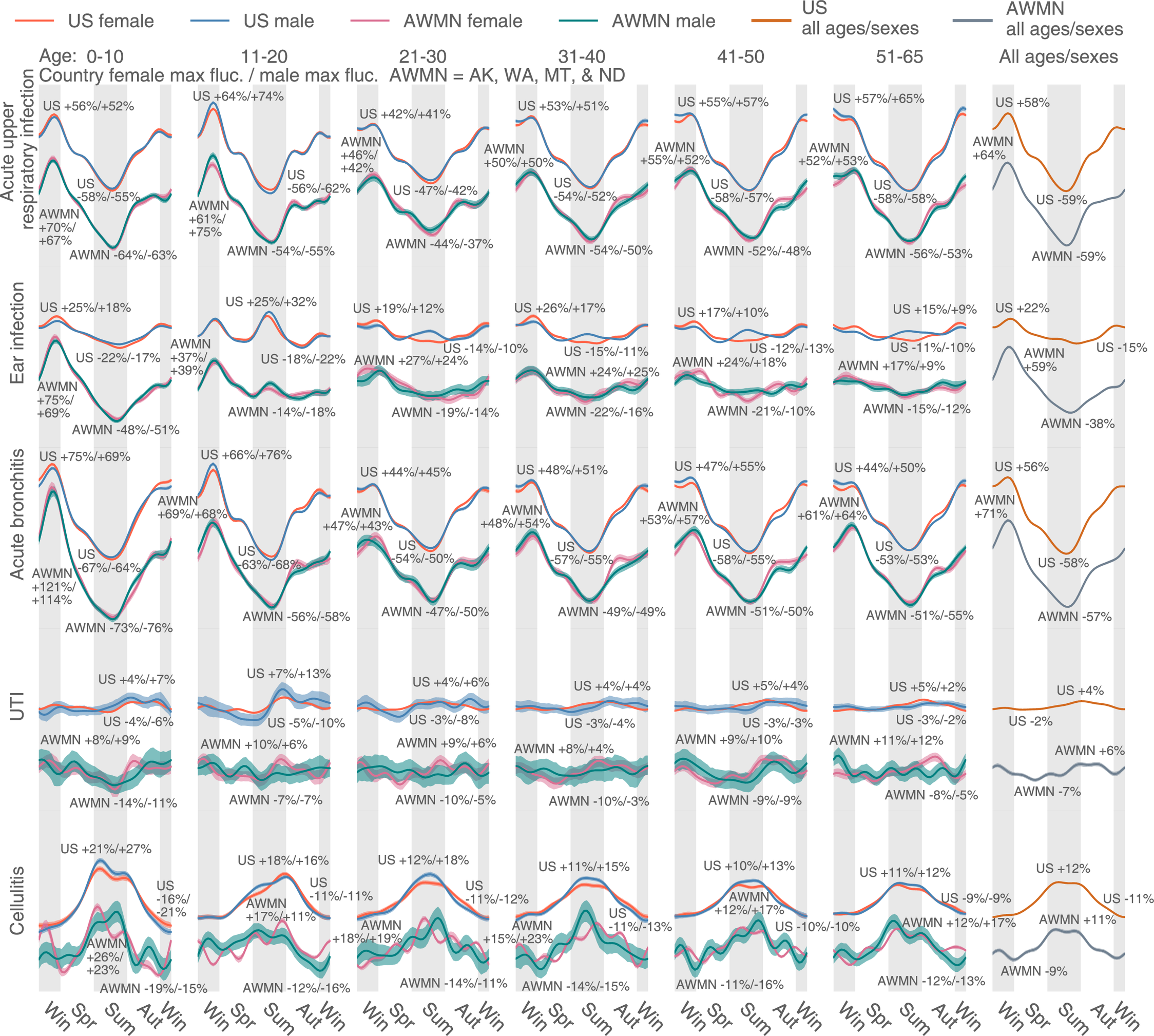

Supplement: S4 Fig — The data underlying this figure can be found in https://doi.org/10.5061/dryad.vdncjsxv6. AK, Alaska; MT, Montana; ND, North Dakota; WA, Washington. (TIF) [file pbio.3001347.s014.tif]

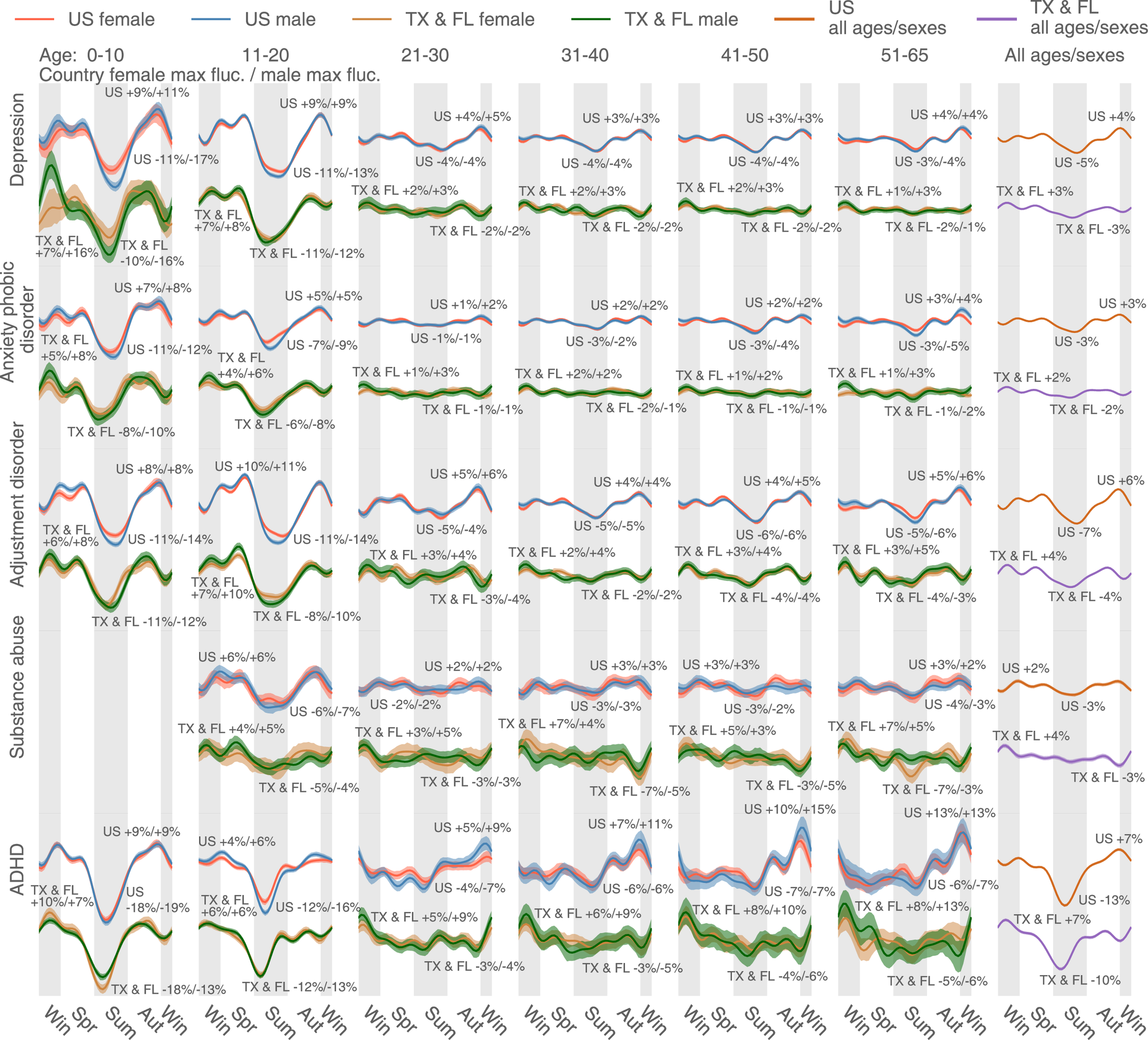

Supplement: S5 Fig — The data underlying this figure can be found in https://doi.org/10.5061/dryad.vdncjsxv6. FL, Florida; TX, Texas. (TIF) [file pbio.3001347.s015.tif]

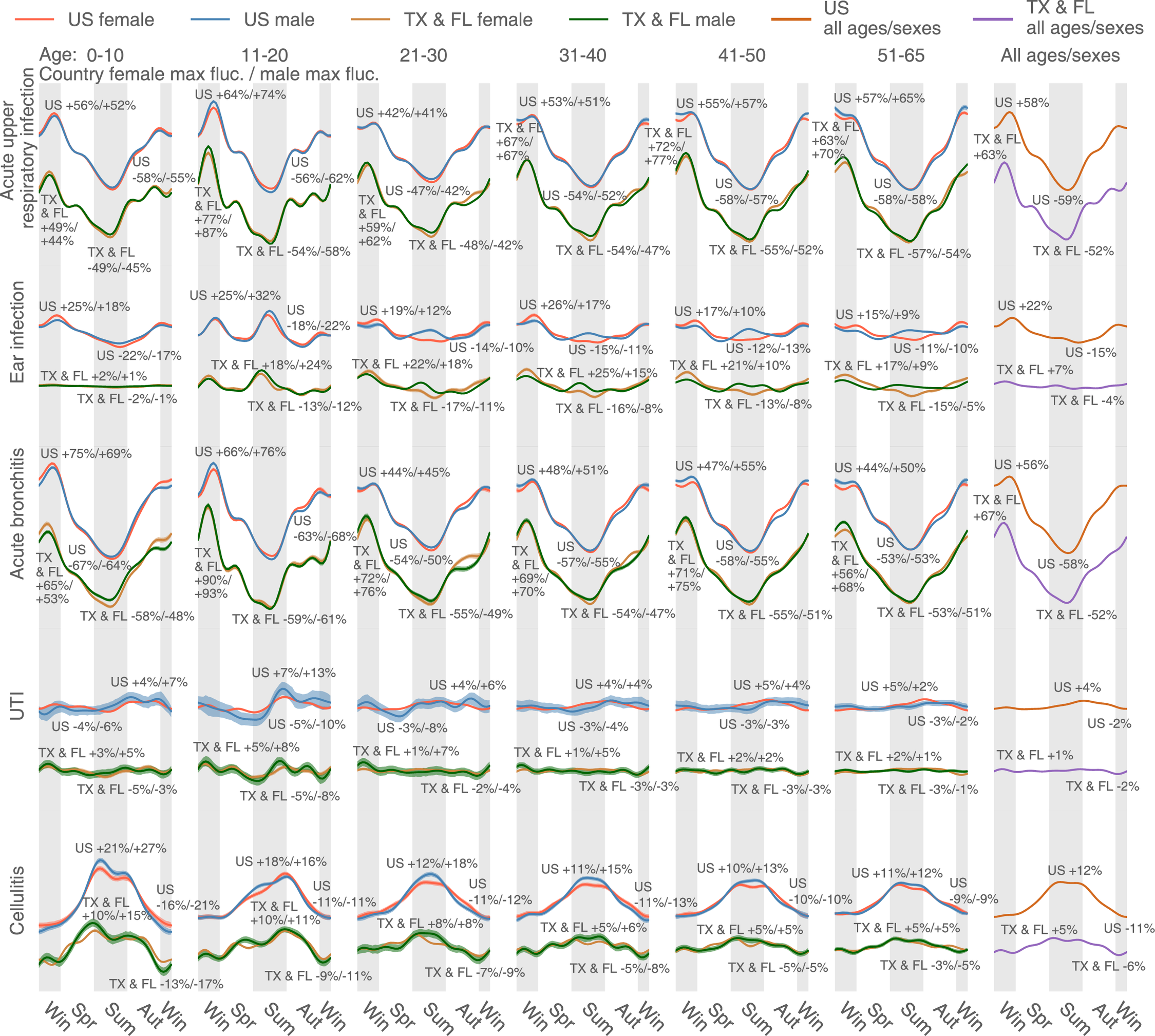

Supplement: S6 Fig — The data underlying this figure can be found in https://doi.org/10.5061/dryad.vdncjsxv6. FL, Florida; TX, Texas. (TIF) [file pbio.3001347.s016.tif]

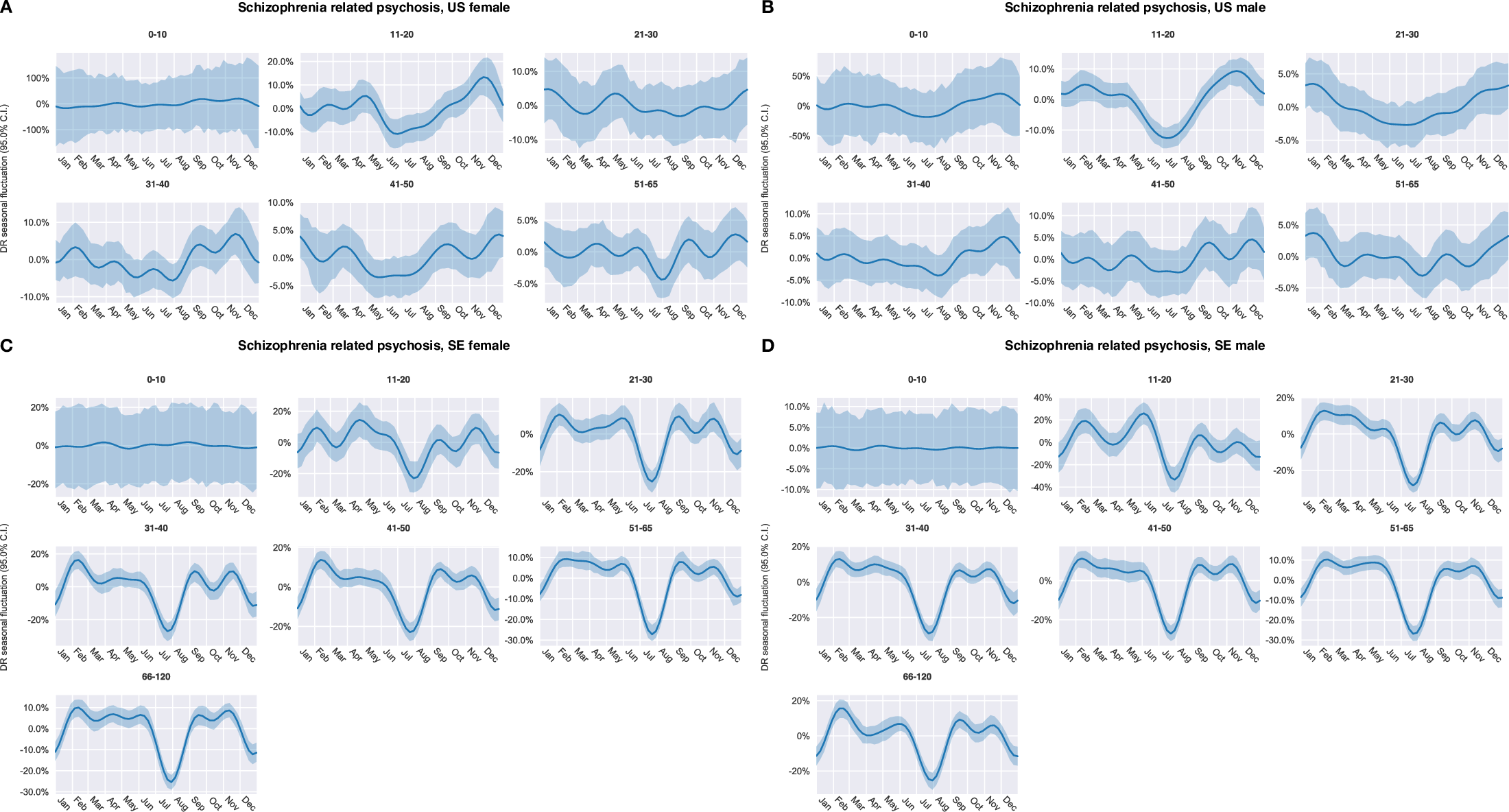

Supplement: S7 Fig — The data underlying this figure can be found in https://doi.org/10.5061/dryad.vdncjsxv6. SE, Sweden. (TIF) [file pbio.3001347.s017.tif]

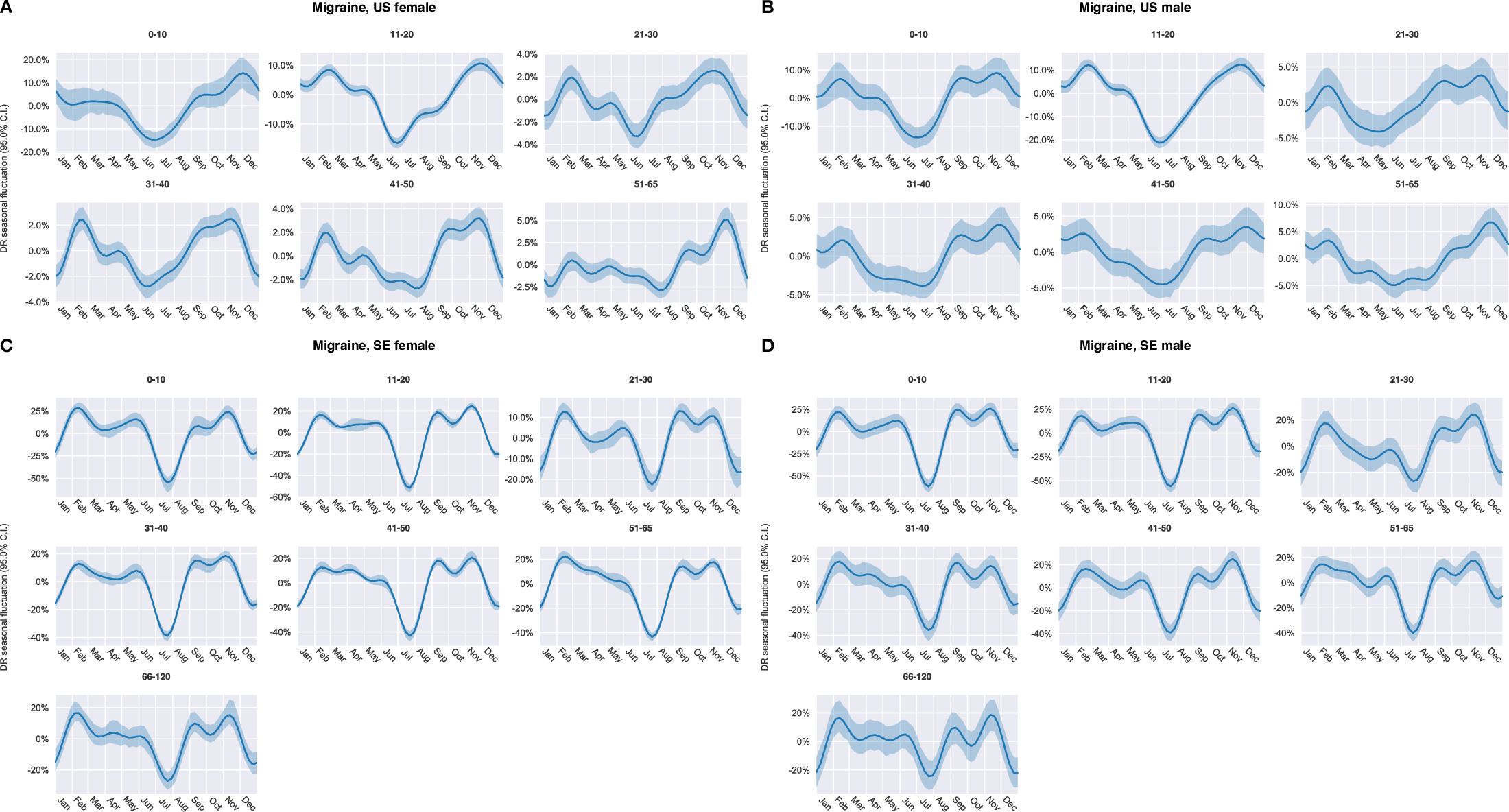

Supplement: S8 Fig — The data underlying this figure can be found in https://doi.org/10.5061/dryad.vdncjsxv6. SE, Sweden. (TIF) [file pbio.3001347.s018.tif]

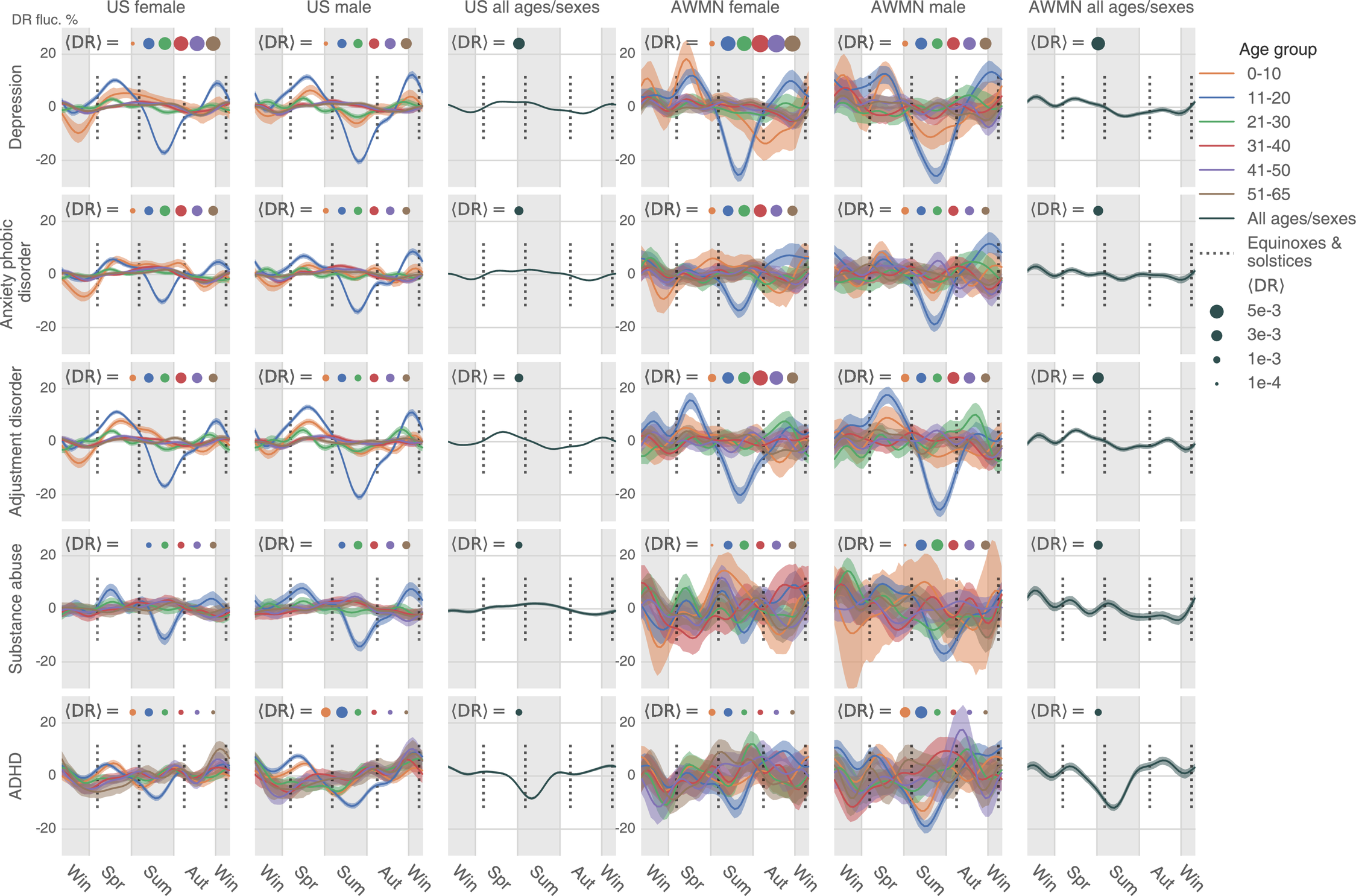

Supplement: S9 Fig — The data underlying this figure can be found in https://doi.org/10.5061/dryad.vdncjsxv6. AK, Alaska; MT, Montana; ND, North Dakota; WA, Washington. (TIF) [file pbio.3001347.s019.tif]

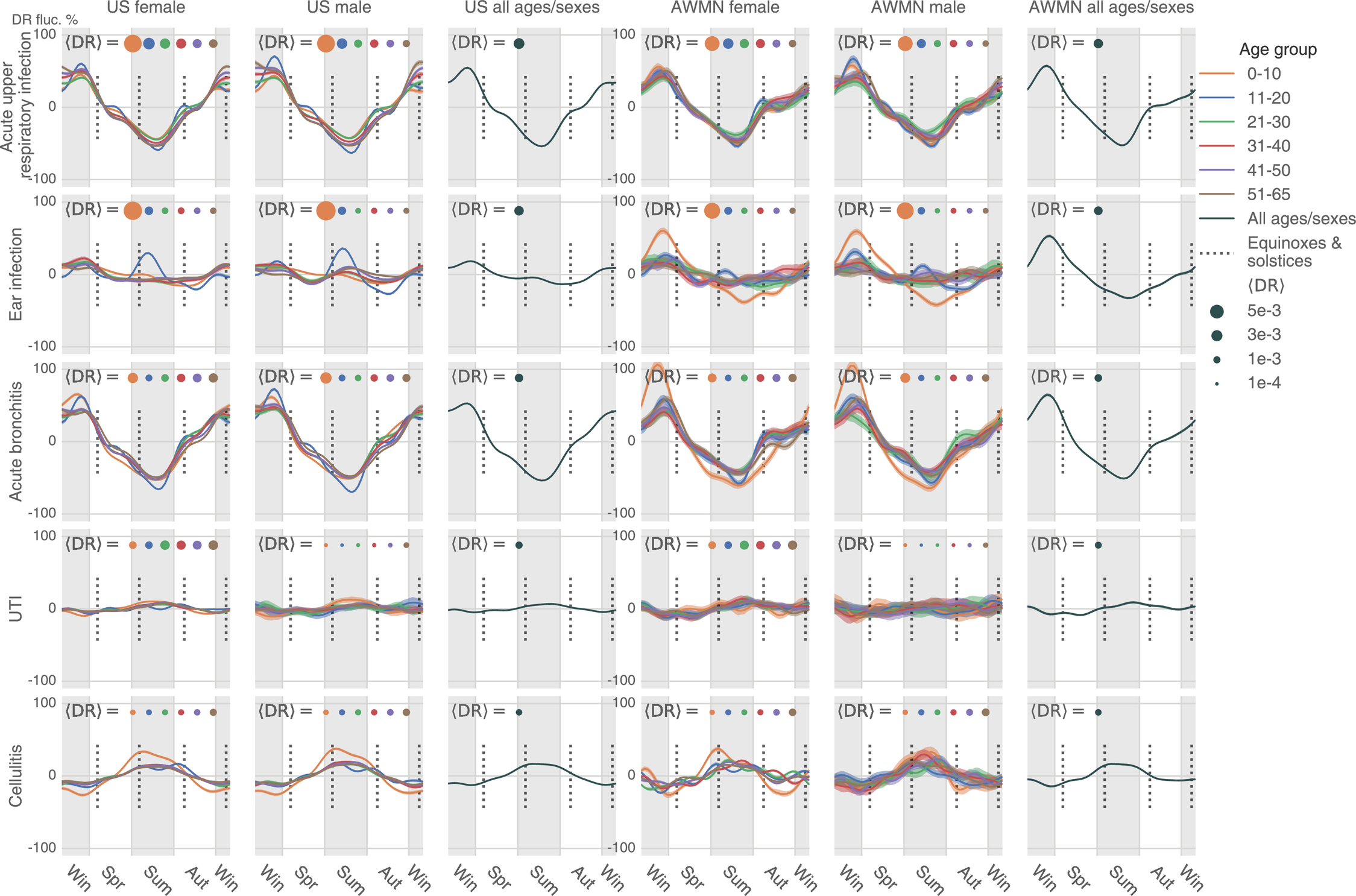

Supplement: S10 Fig — The data underlying this figure can be found in https://doi.org/10.5061/dryad.vdncjsxv6. AK, Alaska; MT, Montana; ND, North Dakota; WA, Washington. (TIF) [file pbio.3001347.s020.tif]

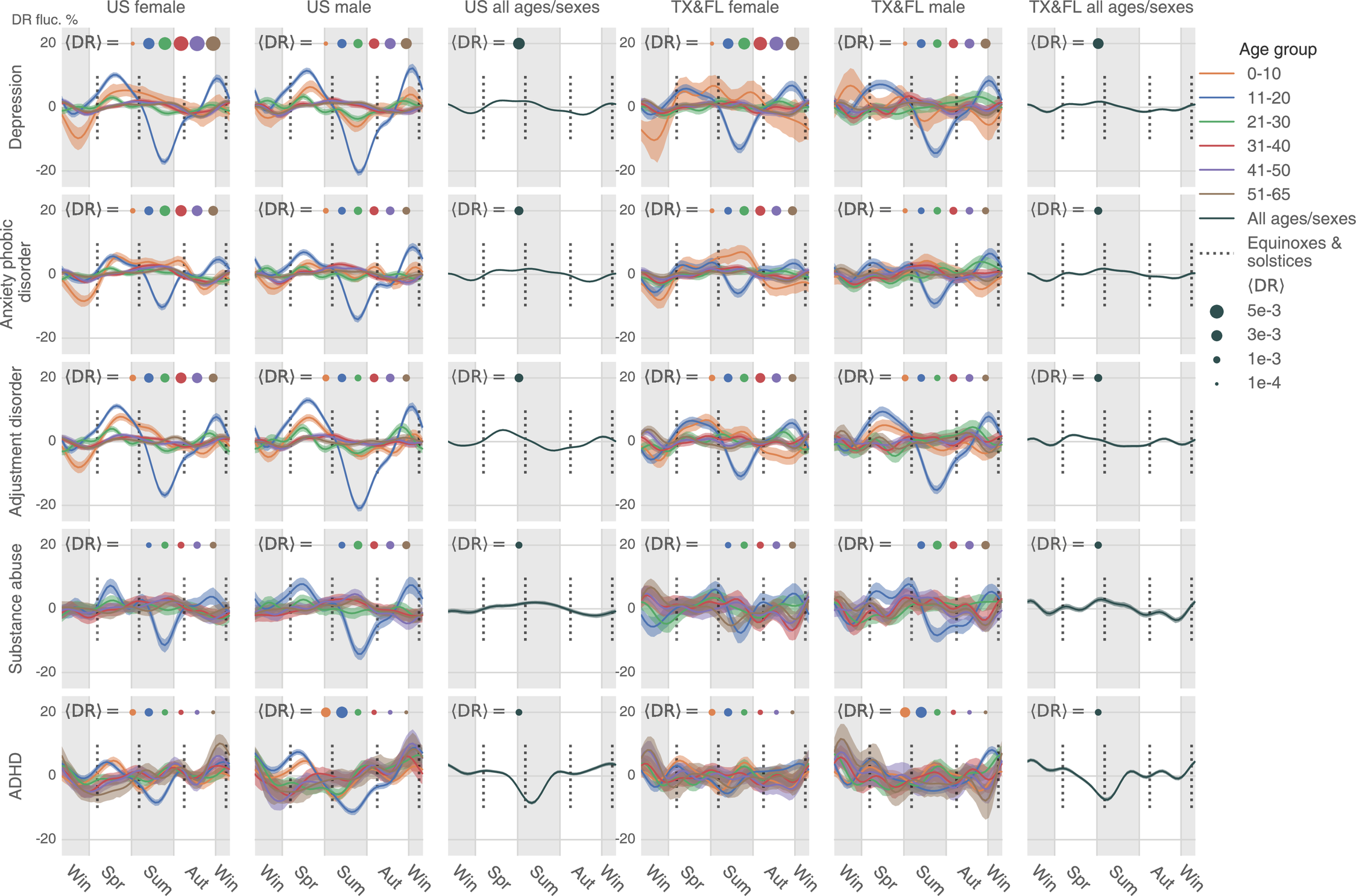

Supplement: S11 Fig — The data underlying this figure can be found in https://doi.org/10.5061/dryad.vdncjsxv6. FL, Florida; TX, Texas. (TIF) [file pbio.3001347.s021.tif]

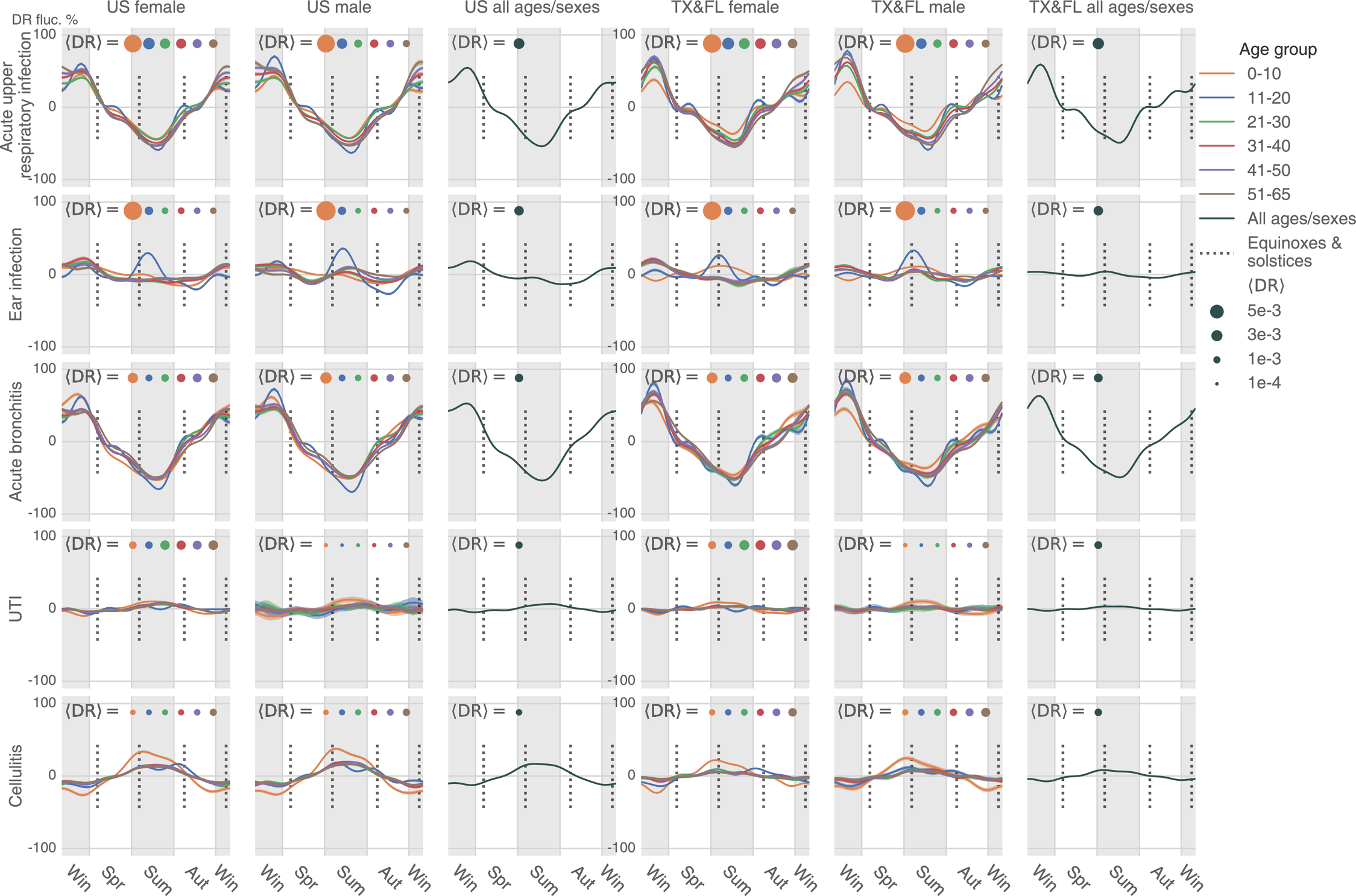

Supplement: S12 Fig — The data underlying this figure can be found in https://doi.org/10.5061/dryad.vdncjsxv6. FL, Florida; TX, Texas. (TIF) [file pbio.3001347.s022.tif]

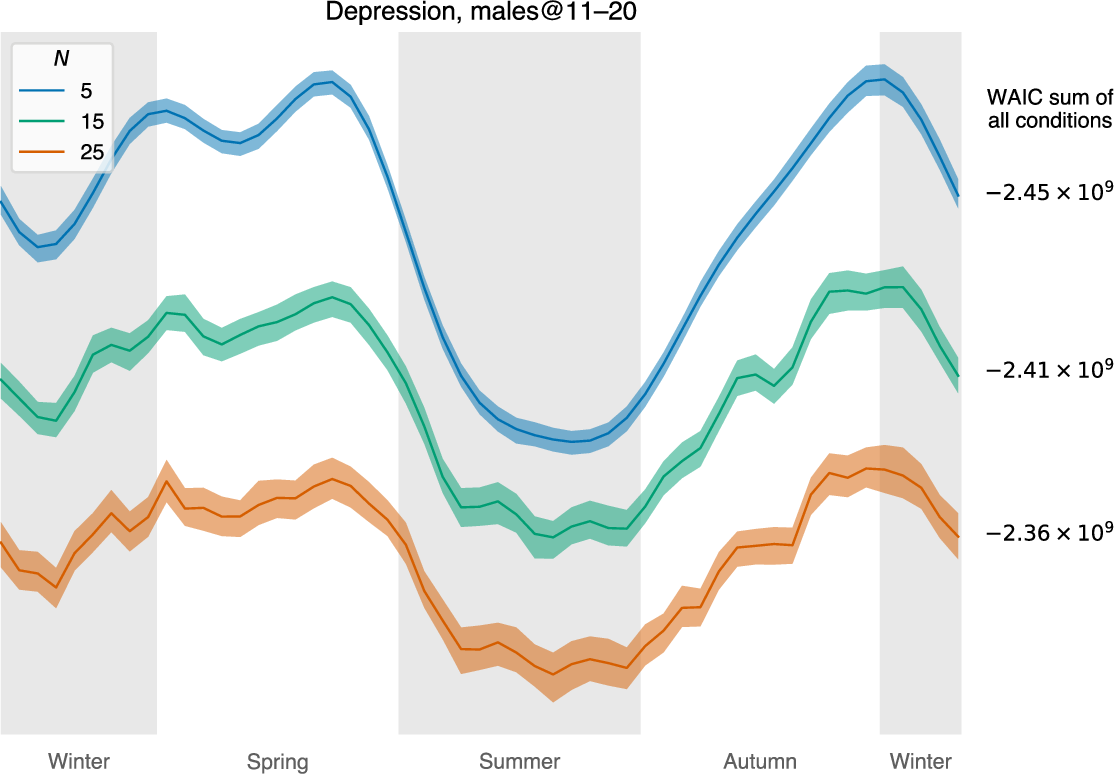

Supplement: S13 Fig — The model with N = 5 has the lowest sum of WAIC over 33 psychiatric and 47 infectious diseases. It suggests the simpler model is good enough to model disease seasonality. In the example of depression in young males, adding up harmonics would not help the estimation, given the intrinsic simplicity of seasonality. The data underlying this figure can be found in https://doi.org/10.5061/dryad.vdncjsxv6. WAIC, Watanabe–Akaike information criteria. (TIF) [file pbio.3001347.s023.tif]
