# Supplementary material for: Do psychiatric diseases follow annual cyclic seasonality?
Source: PLoS Biol. 2021 Jul 19;19(7):e3001347. doi: 10.1371/journal.pbio.3001347 (PMC8345894; doi:10.1371/journal.pbio.3001347)
Supplement: Attachment — Submitted filename: Response_to_Reviewers_V5.docx [file pbio.3001347.s024.docx]

Reviewer #1:

    This paper uses two large healthcare datasets to examine temporal fluctuation in major psychiatric conditions in the USA and Sweden. Despite the impressive datasets and statistical analyses performed there are a number of critical conceptual problems with the paper, as follows:

    1.  The hypothesis and title are poorly articulated. The research question in the title is not directly answered in the paper, though does present evidence that the cyclical patterns of psychiatric disorders are very different to infectious diseases. If I interpret their results correctly, psychiatric disorders (or hospitalised diagnoses therefore) are more patterned than infectious diseases. The hypothesis underlying the article is further based on conjecture that any patterns are due to the "annual light-dependency cycle" but this cannot and is not tested in the paper (the paper lacks data on individual or even ecological exposure to sunlight hours (which would also vary by latitude in both Sweden and the USA)) or, moreover, the circadian cycles on which the discussion speculates. The patterns may be driven by a variety of other causal effects - most of which are equally untestable - the role of vitamin D, for example, or changes in exercise levels. As a result, I feel the paper reaches in places.

We are grateful to the reviewer for pointing out these limitations in our manuscript. In the revised manuscript, we have made an effort to clarify our hypothesis and the central conclusion. We also strived to articulate whether specific conjectures are testable with data accessible to our study, and what additional data would be required to test them. Our revised title is as follows:

*Probing Annual Disease Incidence Cycles in US and Sweden*

Our hypothesis was that patients with psychiatric conditions have common patterns of annual health-seeking behavior.

    2.  I am not an expert on the US data, but have worked with Swedish register data on a regular basis. The paper does not provide sufficient detail on how the diagnostic data and cohort was determined from the National Patient Register. For example, did the study only consider the primary diagnosis at each visit, or any of up to 20 (or more) diagnostic codes which may be made at a single visit. Did the analyses take into account the clustering of repeated diagnoses ("relapses") within individuals, and how did the authors distinguish between spells of care for the same episode, from a relapsed episode? I am unaware of any definition of remission which is possible from using the NPR, but the paper mentions they were interested in remission, so I am curious how this was operationalised in Sweden (and for that matter in the US data - was it simply the absence of diagnosis?).

We have significantly expanded our methodological description to include all the necessary details. We analyzed all codes associated with the same visit, in both datasets. The visits were analyzed in weekly resolution.

We could not distinguish between the first incidence and relapses in the US data because of the lack of complete health history, while it is possible to do so in the Swedish data. All visits and codes, including the repeated diagnoses, were considered in our analysis. Therefore, our seasonality results are composed of both the first incidences and relapses of conditions. We kept using the terms “diagnosis” and “diagnoses” but not “prevalence” or “incidence” to avoid misinforming about the analysis and results – they were are purely regarding diagnoses.

    3.  The NPR in Sweden is not suitable for studying depression, as most cases of depression do not get treated in secondary care, but in primary care, not covered by the register. This seems like the generalisability of the findings may be limited to the severest cases.

The reviewer is correct. The NPR in Sweden covers psychiatric specialty care, but not primary care where most patients with depression get treated. Depressive patients remitted to psychiatric specialty care are those with treatment-resistant depression or severe depression with psychotic symptoms, increased risk of suicide, severe dysfunction (patient does not get out of bed, does not eat), suspected bipolar disorder, etc. We have revised the manuscript to make this point clear.

    4.  The apparent patterns in Sweden may be entirely attributable to artefact of the Swedish holiday system where during the summer months, a substantial proportion of the population go on vacation to summer homes and so forth (between June-August). This seems entirely possible to account for the lower levels of diagnoses made during this period, and likewise the dip in the winter months for several psychiatric conditions around Christmas and New Year, which would seem to further argue against a circadian hypothesis. The peak months for most psychiatric conditions are spring and autumn, presumably coinciding with periods when health services are operating closer to their nominal workforce capacity.

We are thankful to the reviewer for raising this very valid point. In the revised manuscript, as per your suggestion, we used all medical visits (for all conditions or diseases) as our control, and compared a raw seasonality function $s(t), t\in[0, 365.25/7\mathrm{week}]$ to the seasonality of all conditions $s_{\mathrm{all}}(t)$:

$$s_{\mathrm{corrected}}\left( t \right)=s\left( t \right)-s_{\mathrm{all}}(t)$$

This helps us to exclude potential artefacts due to holidays and other socioeconomic activities. After correcting the seasonality, we still found seasonal variation in diagnosis rates in some psychiatric diseases and infections.

    5.  Seasonal effects for schizophrenia have been consistently demonstrated, but these are associated with a Winter-Spring birth and effects on neurodevelopment, not the factors immediately precipitating onset/remission. It feels like a missed opportunity to have delved deeper into this issue.

We have added more discussion about the seasonal effects for schizophrenia in the revised manuscript. Unfortunately, the US data comes with only year of birth information for all enrollees. Due to this data limitation, it is not possible for us to ascertain the seasonality of birth for all enrollees.

    6.  Many elements of the methodology appear in the results section and this interrupts the presentation of the data.

We have significantly revised our results and methodology sections to improve the readability and flow of the manuscript. Specifically, we added a new plot (Fig 2) to better explain the methods and procedures in the main text. We also added more discussion explaining our model design and how we reached the results and plots.

    7.  In the abstract I am unclear what "seasonal affective depression" means - the authors seem to have conflated two diagnoses. 

We apologize for the typo. We have modified all such phrases to “seasonal affective disorder” in the revised text.


Reviewer #2:

    The main problem with the paper is the inability to properly rule out illness behaviour as the main driver of the seasonal effects. This is difficult to do, however one specific strategy I would implement is to replace infectious diseases as the comparator, instead using miscellaneous chronic medical illnesses instead. Acute infectious diseases are the worst possible comparator for this purpose because they are the least likely disorders to be driven by illness behaviour..rather they are almost 100% driven by seasonal biological realities. Chronic medical conditions on the other hand would offer an excellent control for general illness behaviour and other non-specific effects that might have a seasonal basis.

    Further to this point, more consideration of overall seasonal patterns across all psychiatric and medical diagnoses would be of interest....for example I wondered whether the data could be presented as ratios, using total medical visits as the denominator. Truly seasonal disorders should have a greater proportional prevalence relative to all disorders across seasons....using total visits as a control would help in this way.

We thank the reviewer for this valuable suggestion. In the revised manuscript, as per the reviewer’s suggestion, we utilized all-diseases (or conditions) as a baseline to correct the seasonality of test groups for comparative analysis (Fig 2 and S1 Fig). To correct a yearly seasonality function $s(t), t\in[0, 365.25/7\mathrm{week}]$, we subtracted the seasonality of all conditions $s_{\mathrm{all}}(t)$:

$$s_{\mathrm{corrected}}\left( t \right)=s\left( t \right)-s_{\mathrm{all}}(t)$$

Therefore, if we assume that a non-seasonal disorder would show a yearly periodic fluctuation the same as the all-condition baseline, the corrected seasonality will tell us how much more or less a disease deviates from our expectations. The results from this additional (corrected) analysis can be found in results section under ‘Corrected Seasonality Analysis’ heading.

The argument that holiday patterns are different across locations is not strong....many people change their lifestyle and priorities when the weather is nice whether or not on vacation.

In the revision, we’ve discussed more about how lifestyle, priorities, and other socioeconomic activities may vary with seasonal and weather changes, which is reflected in the holiday patterns.

    The authors should mention that school/work pressure in the fall may explain the ADHD seasonal effect. They should also discuss a now solid group of studies showing that ADHD patients have high rates of seasonal depression and phase delays in sleep rhythms.

Thank you for the suggestions! We have added more discussion about how academic pressure and holiday relief affect the ADHD seasonal patterns, and the connection between ADHD and seasonal depression/phase delays in sleep rhythms.


Reviewer #3 [identifies herself as Micaela E. Martinez]:

    Dear Authors and Editors,

    I enjoyed reviewing the submitted manuscript "Are Psychiatric Disorders as Seasonal as Infections?" by Zhang et al. In this manuscript, the authors use two extensive datasets to test for seasonality in psychiatric diseases. The data were quite striking and the results clearly demonstrated seasonality among psychiatric diseases. The analysis was relatively straightforward and their results clearly communicated. The conclusion that psychiatric diseases (specifically ADHD, substance abuse, adjustment disorder, anxiety phobic disorder, and depression) are elevated in the spring, autumn, and winter has great public health importance. I have provided comments below for additional detail and discussion that I believe would be needed before publication. 

    Overall, this study is novel with significant findings and has an appropriate statistical design. It is not clear to me if the data and example code needed to replicate the analysis are provided. The authors should indicate if their aggregated time series will be placed on Dryad or another digital repository. 

    Sincerely,
    Micaela E. Martinez, Columbia University
    (I provide non-anonymous review in support of double-open or double-blind review protocols)

Thank you for reviewing the article! We have provided the data, code, and results on our project’s web site <https://github.com/hanxinzhang/seasonality>.

    Major comments.

    It would be helpful to explain why these five particular psychiatric diseases were chosen. Were these the most prevalent in your datasets?

We chose the five psychiatric diseases and five infectious diseases that were the most prevalent in our data. We explained this in the main text (Line 252).

    It would be helpful to see the seasonal curves for the five diseases on Fig 1 all on a single plot in order to see the similarities in the seasonality.

We tried to present all Fig 1’s (now Fig 3) seasonality curves on a single plot but that resulted in a very busy plot where it was difficult to determine which trend represents which group and disease. Thus, we decided not to include that complicated and busy plot. We believe it is clearer to show the similarity of seasonality across diseases by stacking the curves as in Fig 3.

    It would be helpful to see the raw data and posterior curves for each psychiatric disease with data from all age groups and both sexes aggregated (i.e., similar to the raw observations shown in Figure S8, Panel D left and the posterior expectation on the right). 

For each studied disease, we created a plot that demonstrates how well our model fit the raw data. These plots can be found on the project’s web site (<https://github.com/hanxinzhang/seasonality/tree/master/results>) under the “posteriors” directory for each test district (the US, Sweden, etc.). For example, the following figure shows the posterior expectation and corresponding credible intervals (HPD intervals) for females’ adjustment disorder based on all data in the US, compared to the raw data. Note that, for this particular condition, the credible intervals (shown in green shadow) are very narrow and almost invisible because the disease is very common.

    For the psychiatric diseases, it would be helpful to see the seasonality on a polar coordinate where the summer solstice, winter solstice, autumn equinox, and spring equinox are marked so we may clearly see the seasonal pattern relative to photoperiod extremes and transitions.

Thank you for the suggestion. We now include solstice and equinox points on our plots (Figs 6 and 7, S9-S12 Figs) in the revised manuscript.

    It would be helpful to explore the diagnosis rate relative to daylength in each country. The seasonal change in photoperiod is much more extreme in Sweden than the US (and this could be an important feature, as discussed by the authors) and a formal analysis of the daylength effect could be done for the supporting information.

We appreciate the suggestion: The analysis suggested by the reviewer was performed as a part of a separate study, specifically looking at the association between latitude/sunlight cycle and disease seasonality.

    line 79. It reads as though the data will be analyzed in 4 seasonal bins, whereas, the seasonal curves are actually in a daily or weekly resolution (from what I can infer). It is important to note that the 4 seasons are being used as reference points for discussion and not for aggregating the data into seasonal bins. It will also be important to note if the time series were daily or weekly. I am assuming daily based on the holiday effect mentioned in Fig S8.

We now made this clear in the revised manuscript that our seasonal analysis was performed entirely in weekly resolution and the four meteorological seasons are simply for reference and discussion purposes.

    line 105. Following on the comment above, are the curves in Fig 1 derived from daily data? Because above you only mention the 4 seasonal windows for the data. The methods suggest daily or weekly resolution.

Our seasonal analysis was performed entirely in weekly resolution.

    Fig 1. Again, adding to the comment above, since the results come before the discussion, please describe how the curves are derived. I am assuming the analysis is using weekly or daily data because there is such a great discrepancy between min and max values for some curves. For instance, the max is 1/2 the min for depression in Sweden for 11-20 yr olds.

In the revised manuscript, we have added a new section and new plots (Figs 1 and 2) in the main text to better explain how we analyzed the data, corrected confounding factors, and inferred seasonality. Again, all analyses were performed in weekly resolution.

    Fig 1. It would be interesting to include a diagnosis that doesn't have a biological basis as a "control". Something like injuries from accidents - we wouldn't expect to be seasonal. Otherwise people may argue that the observed seasonal patterns are due to less healthcare seeking behavior in summer.

Thank you for the suggestion. We performed additional analysis by using all medical visits (all conditions) as our control, assuming the general seasonal pattern of all medical visits reflects the overall socioeconomic fluctuation. Please see the full details of this additional analyses in the revised manuscript.

    Fig S3. For all of the figures showing the seasonality, such as Fig 1 and Fig S3, it would be helpful to have a y-axis scale to tell which age group has the highest incidence. For instance, in Fig S3 is the seasonality of ear infection simply more detectable in the youngest age group because this is the group with the highest incidence of ear infection? If there were a y-axis scale, the reader would be able to compare the incidence across age groups.

To compare the incidence rate across age groups and diseases, we can simply refer to the time-average diagnosis rate $\left\langle\text{DR} \right\rangle$ marks on the corrected seasonality figures (Figs 6, 7 and S9-12 Figs). The time-average diagnosis rate is the same for the uncorrected result. For ear infection specifically, the 0-10 age group does have the highest diagnosis rate, but this does not hold for other diseases. For psychiatric diseases in the US, such as depression, the older age groups have higher diagnosis rate as indicated on Fig 6, but the seasonal variation is larger in younger groups (Fig 3, 0-10 and 11-20). We chose not to impose a y-axis scale in order to avoid complicating our plots. Additionally, this presentation style places a stronger emphasis on relative fluctuation, as compared to the disease’s own average trend.

    line 231. As mentioned above, it would be good to have a 'negative control', something you don't expect to be seasonal because it doesn't have a biological basis, such as injury from accidents.

As mentioned above, we performed additional analyses by using all medical visits (and all conditions recorded at the same visit) as our control. We did not use a particular disease as the control because conditions like injuries could also be seasonal. Many injuries are the result of traffic accidents that may also bear some degree of seasonality. For example, the governmental report in 2005 showed that the total vehicle occupant fatalities are higher in summer: <https://crashstats.nhtsa.dot.gov/Api/Public/ViewPublication/809855>.

An infectious disease, in its acute manifestation, requires the immediate attention of a physician. Most infectious diseases are transient (aside from a few chronic examples, such as malaria, AIDS, and herpes). Therefore, we expect that annual encounter rates for healthcare seeking patients suffering with infections reflect real seasonality rather than exclusively annual patterns of recreational activities and vacations.

The situation might be different with patient visits associated with care for chronic diseases, elective procedures, and routine health checkups; harsh weather and vacation time may delay a visit and may require adjustment in analysis. Therefore, we produced two versions of analysis of annual rates of disease-related visits: “uncorrected” and “corrected.” The former type of analysis answers the question “How healthcare-seeking visits of patients with disorder *X* are distributed across seasons?” The latter type of analysis answers a different question: “How is *the proportion* *of* healthcare-seeking visits of patients with disorder *X* *with respect to all healthcare-seeking visits for this interval of time* distributed across seasons?”

First, we argue that the psychiatric conditions we chose to examine in this study behave more like acute infections rather than elective procedures. For example, depression cases recorded in the Swedish registries are the most severe, where the patient needs immediate hospitalization (for example, because she stops eating). Milder depression cases, where a patient requires an antidepressant prescription, are handled by primary care providers and do not show up in health registries.

Second, our uncorrected seasonality results better resonate with published smaller-scale observations than our corrected results: Past studies report anxiety and depression rates higher during the spring than summer in both Europe [1] and the US [2]; Bipolar disorder symptoms receded in summer in patients in Arctic areas of Northern Fennoscandia [3], and; Depression and suicide in the US were reported to be higher in the spring than in the summer [4]. An increase in substance abuse-related admissions to hospitals was also observed in the spring compared to summer in Vietnam [5]. Studies in Vietnam have also reported that mood disorder-related hospital admissions were higher in the spring and fall compared to the summer, in agreement with our uncorrected seasonality results [5]; in corrected results a subset of age groups displayed higher rates of depression in the summer than in the fall, see Figures 3 and 6.

Third, our attempt to “correct” the individual disease seasonality rates by adjusting for the total rate of healthcare-seeking events (see Figs 6 and 7) produced nonsensical results. For example, while uncorrected rates of substance abuse in Sweden [6, 7] have been reported to be higher in the spring than in the summer (and this is what our uncorrected seasonality shows), in the corrected Swedish results, the substance abuse rate completely reversed this trend, with the disorder rate higher in the summer than in the spring. In the US, the corrected data trends also changed drastically with respect to the uncorrected trends – for psychiatric conditions, only one age group (eleven to 20-year old patients) preserved their behavior across all psychiatric conditions with compared to uncorrected seasonality results. The remaining age groups acquired additional idiosyncratic properties that are not aligned with anything known about these diseases.

1.	Sebestyen B, Rihmer Z, Balint L, Szokontor N, Gonda X, Gyarmati B, et al. Gender differences in antidepressant use-related seasonality change in suicide mortality in Hungary, 1998-2006. The world journal of biological psychiatry : the official journal of the World Federation of Societies of Biological Psychiatry. 2010;11(3):579-85. Epub 2010/03/12. doi: 10.3109/15622970903397722. PubMed PMID: 20218927.

2.	Postolache TT, Langenberg P, Zimmerman SA, Lapidus M, Komarow H, McDonald JS, et al. Changes in Severity of Allergy and Anxiety Symptoms Are Positively Correlated in Patients with Recurrent Mood Disorders Who Are Exposed to Seasonal Peaks of Aeroallergens. Int J Child Health Hum Dev. 2008;1(3):313-22. Epub 2008/01/01. PubMed PMID: 19430577; PubMed Central PMCID: PMCPMC2678838.

3.	Pirkola S, Eriksen HA, Partonen T, Kieseppa T, Veijola J, Jaaskelainen E, et al. Seasonal variation in affective and other clinical symptoms among high-risk families for bipolar disorders in an Arctic population. Int J Circumpol Heal. 2015;74. doi: ARTN 29671

10.3402/ijch.v74.29671. PubMed PMID: WOS:000369579600001.

4.	Schwartz PJ. Chris Cornell, the Black Hole Sun, and the Seasonality of Suicide. Neuropsychobiology. 2019;78(1):38-47. Epub 2019/03/29. doi: 10.1159/000498868. PubMed PMID: 30921807; PubMed Central PMCID: PMCPMC6549453.

5.	Trang PM, Rocklov J, Giang KB, Nilsson M. Seasonality of hospital admissions for mental disorders in Hanoi, Vietnam. Glob Health Action. 2016;9:32116. Epub 2016/08/28. doi: 10.3402/gha.v9.32116. PubMed PMID: 27566716; PubMed Central PMCID: PMCPMC5002036.

6.	Bradvik L, Berglund M. Seasonal distribution of suicide in alcoholism. Acta Psychiatr Scand. 2002;106(4):299-302. Epub 2002/09/13. doi: 10.1034/j.1600-0447.2002.02234.x. PubMed PMID: 12225497.

7.	Levine ME, Duffy LK, Bowyer RT. Fatigue, Sleep and Seasonal Hormone Levels: Implications for Drinking Behavior in Northern Climates. Drugs & Society. 1994;8(2):61-70. doi: 10.1300/J023v08n02_04.

    lines 233-239 . As mentioned above, it would be important to also address the difference in incidence among age groups. For example, in Sweden, the trough in ear infection in 0-10 is in the summer. I would expect that a large fraction of ear infection occurs in the 0-10 yr old group; thus, is the seasonality in this group the most "representative" seasonality for ear infection? It might be that the ability to detect the seasonality in some age groups is limited due to low incidence and therefore it is important to show the actual number of cases in each group, or cases per 100,000. Following on my comment above, it would be good to show the curve for each disease where males and females and all age groups are aggregated.

In Sweden, age zero to ten is the most representative group for ear infection, as it has the highest crude incidence rate. We have created new plots to show the absolute change in incidence rates for each disease discussed in the main text, aggregating all age groups (Figs 6, 7 and S9-12 Figs, the all ages/sexes columns).

    Discussion section. It is necessary in the discussion section for the authors to discuss how circadian rhythms relate to seasonality, because it will be confusing for many readers. Perhaps the authors can reference more of the literature on SAD and also the literature on melatonin seasonality (e.g., papers by Thomas Wehr and by Ken Wright).

We have added more details to explain how circadian rhythms and daylight could be related to seasonality in the revised manuscript.

    Discussion section. It would also be helpful for the authors to provide some biological information about the psychiatric disorders in this study so readers can have a better understanding of how clocks could play a role. Are these disorders impacted by hormones, the immune system, sleep, or metabolism? Also, it could be good for the authors to discuss seasonal light exposure in general and how this may impact circadian rhythms.

In the revised Discussion section, we’ve provided more information about the biological mechanism of circadian rhythms and their link to psychiatric disorder incidents.

    line 435. This paragraph needs more explanation. The way it reads to me is that the parameters for the trend, shifts, and noise terms were estimated and then the seasonal remainder was estimated last. Is this correct? Or were all parameters estimated simultaneously? Or estimated in a specific order? When it says the authors "divided the raw DR seasonality to its average DR over 570 weeks", does this mean they took the seasonal component from Eq 1 and divided it by the linear trend + yearly shift from Eq 1? Or that they took the seasonal component and divided it by the mean of the yearly trend + yearly shift across all years?

We estimated all parameters, trend, shifts, seasonality, and noise terms simultaneously under the same hierarchical model. However, because we assigned the harmonic parameters $p_{i,j,n}$ and $q_{i,j,n}$ for each population stratum $i\in\{1,2,\ldots,100\}$ of age group $j$, the raw estimation (post hoc) for seasonality should be the weighted sum of all strata, as shown in Expressions (15) and (16). Here, a population stratum is a group of patients who joined and left our data at approximately same time.

To estimate the relative fluctuation in percentage scale, we then divided the raw seasonality estimate by this disease’s (time) average DR. The average DR is the raw observed diagnosis rate from week 1 to week 570:

$$\text{time}\text{-average DR}=\left\langle\text{DR} \right\rangle=\frac{1}{570}\sum_{w=1}^{570} \mathrm{DR}_{w}$$

We noticed that most disease diagnosis rates have an upward trend with respect the first date recorded in the dataset (January 1, 2003). Therefore, we need a time average as the comparator; if we simply divided a disease’s diagnosis rate by the time-sensitive trend, its relative fluctuation varies across years.

lines 441. As mentioned above, due to the age group trends, it would be important to calculate a prevalence/incidence estimate. For example, calculate diagnosis per 100,000 patients in each age group. The seasonal curves that are most important from a public health perspective are those most representative of the disease. Thus, the childhood curves will be most important for the pediatric diseases and the older age groups will be most important for the diseases that usually manifest in adulthood.

We marked the time-average DR for each disease and age group on our new plots (corrected seasonality, Figs 6 and 7, S9-12 Figs).

    lines 450. Please provide what the four center panels in figure S8D are showing, which age groups are shown here? Can you also be more explicit about which parameters are shared among the age groups (within a disease)? You say that "The model fits each population in a hierarchical way so that information is shared across populations", but it seems as though each age group has their own trend and seasonality, so what information has been shared/constrained in this hierarchical fitting?

We created a new figure to better explain the working of our model. The four center panels in Figure S8D elaborates that we assigned parameters to each population stratum $i\in\{1,2,\ldots,100\}$ of age group $j$ separately. We sampled all parameters simultaneously. We shared information across age groups and population stratum because they were sampled from the same priors and hyper priors. For example, for the linear trend intercepts $\alpha_{i,j}$ for population stratum $i$, we sampled them from a skew-normal distribution with an age-specific center $\mu_{j}^{\alpha}$, scale $\sigma_{j}^{\alpha}$, and shape $h_{j}^{\alpha}$. These age specific hyperparameters were also sampled from shared Gaussian process hyperpriors that chained them together across age groups, so that close ages would have close center $\mu_{j}^{\alpha}$, scale $\sigma_{j}^{\alpha}$, and shape $h_{j}^{\alpha}$. We discussed this in more detail in our Method section in the revised manuscript.

    Fig S8. It would be helpful to have panels A, B, and D in the main text of the manuscript to give readers a feel for the modeling workflow. Also, panel A needs a better description in the caption. For example, it is not clear what is meant by "naive fitting line". Also, in this panel, shouldn't the "periodic patterns" curve not have a trend in it? I believe it should be flat unless it is illustrating the periodic + trend.

We added a new methodology plot in the main text that provides more mechanistic details of our model and data fitting. We also moved Fig. S8A to the main text and explained the shortcomings in our data and demonstrated how we addressed those in our model.

    Minor comments.

    line 20. "We found that psychiatric diseases' annual patterns are remarkably similar across the studied diseases in both countries, with the magnitude of annual variation significantly higher in Sweden than in the US for psychiatric, but not infectious diseases, potentially pinning the pathogenesis of psychiatric diseases on circadian rhythms." 
    I suggest rephrasing the sentence above. The fact that (for infectious diseases) the magnitude of seasonality doesn't vary between countries does not tell us anything about clock involvement or lack thereof. This is because acute infectious disease seasonality is shaped by the transmission process. I would recommend breaking the above quote into two sentences. Such as: We found that psychiatric diseases' annual patterns are remarkably similar across the studied diseases in both countries, with the magnitude of annual variation significantly higher in Sweden than in the US for psychiatric, but not infectious diseases. The seasonality of psychiatric diseases suggests the pathogenesis of psychiatric diseases may be driven circadian rhythms…[then explain the logic of how circadian rhythms relate to seasonality]

We rephrased the sentence to “We found that psychiatric diseases’ annual patterns were remarkably similar across the studied diseases in both countries, with the magnitude of annual variation significantly higher in Sweden than in the US for psychiatric, but not infectious diseases.” We avoid speculating about circadian rhythms in the abstract and leave the thorough discussion in the main text.

    line 28. The first sentence sounds a bit off-putting because it sounds like the authors are suggesting that psychiatric disorders are mental constructs rather than biological diseases. This could be offensive to some readers.

Thank you for pointing out this! We rephrased the sentence to “Psychiatric illness induces profound suffering and profoundly affects the lives of patients and their loved ones.”

    line 111. I am confused about this web link. Are these the manuscript data on this website? Were the analyses on this website conducted by another research group? If this is the manuscript data, I suggest it be included and described in supplemental info.

We maintain a more comprehensive project web site on GitHub (<https://github.com/hanxinzhang/seasonality>) to exhibit complete data, code, and results for diseases and regions we reported in this manuscript.

    line 227.  The examples "seasonal access to pathogens" and "summer swimming activities" are a bit misleading. It would be better to mention seasonal transmission of infection and host behavior.

We rephrased the sentence to “Seasonal infection waves are driven by typically well-understood factors, such as seasonal transmission of infection, host behaviors, and seasonal variation of host susceptibility to infections.”

    lines 237. This sentence is problematically speculative, especially since the authors don't show that ear infection is caused by outdoor activity. I suggest removing this sentence: 'It is then plausible to impute this dissimilarity between age groups and sexes to different levels of outdoor activities in the summer, which entails, speculatively, that American males are more active in summer than American females whereas the gender gap is minimal in Sweden.'

Thank you for pointing this out! We agree that this description was speculative, therefore, we have removed it from the text.

    line 262-300. I am not sure of the current formatting requirements for plos bio, but the "assumptions, material and methods section", if it will be in the main text, could be reformatted to "materials and methods" with the list of 6 items presented in more of a paragraph style. This would make the materials and methods section read more seamlessly with the other main text sections.

We have reformatted this section to “Materials and Methods” and merged the six assumptions into a more easy to read paragraph.

    lines 282. I recommend removing this example, it reads as ageist and may perpetuate stereotypes "one group can correspond to mostly healthy young factory workers, while another one could comprise aging oil refinery workers."

Thank you for pointing out – we agree that this may perpetuate stereotypes! We removed the inappropriate description.

    Discussion. Following on my comments above, it would be helpful in the discussion and in Fig 1 to mention that the seasonality was modeled with daily or weekly resolution.

In the revised main text, we explicitly mentioned that all our analyses were in weekly resolution.

    line 374. In Eq 7 and 8, please provide more detail of why this specific formulation is being used for the phase and amplitude. Is there a specific constraint enforced by using the p_{i,j,n} and q_{i,j,n} formulations?

We estimated the seasonality using the linear addition (sine-cosine) form of the Fourier series because the linear form is easier for coding and computing. There was no constraint enforced by the p, q formulations.

We showed the amplitude-phase form and the transformation because we assumed that some readers might be more familiar with this form.

    Fig S8. Panel D, right panel - it would be helpful for readers if you indicate the shift happening in 2009, and perhaps show the seasonality curve, instead of just the age trends and the posterior.

In the new methodology plot, we have explicitly indicated the shift, and the seasonality curve.
