# Supplementary material for: Do psychiatric diseases follow annual cyclic seasonality?
Source: PLoS Biol. 2021 Jul 19;19(7):e3001347. doi: 10.1371/journal.pbio.3001347 (PMC8345894; doi:10.1371/journal.pbio.3001347)
Supplement: Attachment — Submitted filename: Response_to_reviewers_6-21-2021.docx [file pbio.3001347.s025.docx]

**Editor’s notes:**

a) You'll see a diversity of opinion among the reviewers here. Reviewer #2 is largely satisfied, but questions whether the suggested approach was the correct one. We believe that because this approach was also suggested by reviewer #3, who is very well-placed to judge, and as she is now broadly happy with the outcome, that it was indeed a valid way forward...

Thank you, accepted.

b) ...however, because of your decision to also present the uncorrected analyses (which we will respect), this has resulted in some confusion, especially because of the somewhat strong language that you use to dismiss the corrected version (e.g. "nonsensical" is a term highlighted by both reviewers #2 and #3). You will see that reviewer #1, who recommended that we now reject your paper, finds this problem to be fatal....

We modified our manuscript to give attention to both analyses.

c) ...to my thinking, the key thing to bear in mind when revising your paper to address these disparate remarks is to make the paper as clear as possible for the reader, while avoiding misleading them. We strongly recommend that you present both sets of results even-handedly, and with full transparency, until the Discussion, when you should present your arguments as to why you believe that the uncorrected analysis better captures the real picture. This would enable readers to "make up their own minds" while allowing you to present your case. We assume that a solid "answer" must await further research.

Accepted; we tried to clarify both ideas and style as much as we could.

d) So: please revise your text with my comments (above) in mind, and asking yourself what reviewer #1 might think. For example, in the Abstract, instead of "Comparing two sets of results in context of published psychiatric disease seasonality studies, we tend to believe that our uncorrected results are likely to capture the real trends, while the corrected results reflect mostly artefacts generated by idiosyncratically fluctuating volumes of patient health-seeking visits across year"  I would suggest something like "In the context of published psychiatric disease seasonality studies, we discuss whether our uncorrected results or the corrected ones are more likely to capture the real underlying trends."

Done as suggested.

e) Because we do not feel that you can reach a solid conclusion, we think that it would be better to keep an interrogative title (perhaps something like "Does psychiatric disease follow annual disease cycles?" which I note is quite similar to your original title). As US and Sweden are namechecked clearly in the Abstract, we don't think you need them in the title.

Accepted and changed as suggested.

f) Please attend to all the remaining requests from reviewer #3.

Done.

g) Because of the extra analyses, we can no longer consider this paper as a Discovery Report; this is now a much more substantial analysis, and should be published as a full Research Article. Please could you change the article type to "Research Article" when you re-submit?

Done.

h) Please could you update your blurb to reflect the revision and the above comments?

Done.

i) Please could you attend to my Data Policy requests below. I note that your raw data are third-party, and I have treated these as for your previous PLOS Biology paper, which I understand used the same datasets. However, we will need the numerical values that are shown in the Figures; the location of these data should be cited clearly in all of the relevant legends.

All data sets (aggregated data) are deposited to GitHub, with the specific address presented in the manuscript.

j) I note that in your previous PLOS Biology paper you included a statement clarifying the ethics situation, namely "The University of Chicago IRB determined that the study is IRB exempt, given that patient data in both countries were pre-existing and de-identified." If correct, could you possibly include an equivalent statement in the current paper?

Done.

As you address these items, please take this last chance to review your reference list to ensure that it is complete and correct. If you have cited papers that have been retracted, please include the rationale for doing so in the manuscript text, or remove these references and replace them with relevant current references. Any changes to the reference list should be mentioned in the cover letter that accompanies your revised manuscript.

Done.

**Reviewer #1:**

Thank you for submitting the heavily revised analyses for re-review. I still have several reservations with the paper, and the corrected analyses lend support to the idea that these patterns are not specific or unique to psychiatric disorders. The paper also has a number of problems including in drowning in data, making it hard to make sense of the results, and problems with the English language. Overall, I do not think there is strong enough evidence to support the conclusions presented, and the manuscript needs parsing into more carefully articulated ideas. I was not invited to review the original submission and feel the work is not strong enough in its current form.

In the latest revision, we modified our discourse and tried to present both sets of analysis (uncorrected and corrected) even-handedly. In the Discussion section, we argue why we believe the uncorrected analysis and results better resemble the reality, but we do not judge, which of the outcomes is closer to truth. We left the verdict to the reader. A reader can make up their own mind by evaluating both sets of results, based on their own experience and understanding of biological and socio-economic mechanisms of psychiatric diseases.

**Reviewer #2:**

It was my suggestion to use total visits as the denominator in a corrected analyses, in order to correct for non-specific seasonal trends in medical visits. Having now seen the results, which are largely described as non-sensical by the authors, I must acknowledge my relative lack of statistical expertise to know whether this approach is valid. This being the case, I would defer to a statistical reviewer who can solve this with much more expertise. I do think non-specific seasonal effects should be controlled for…not sure is this is the right approach.

We do agree that non-specific seasonal effects should be corrected for. However, we also found it was not easy to pick a normalizer that represents the seasonality resulting from non-biological effects. Many diseases, including injuries, are seasonal, and it is not clear whether the socio-economic factors (e.g., seasonal accidents) leading to the condition overlap with socio-economic factors driving seasonality of psychiatric diseases. For this reason, we did not use a specific disease to control for the non-specific seasonal effects. We relied on the counts of visits associated with any medical reason to control the seasonality.

In the revision, we modified our discourse and made a considerable effort to balance presentation of both sets of analysis (uncorrected and corrected) even-handedly. In the Discussion session, we explain why we believe that the evidence and results better support the uncorrected analysis, but we do not make a decisive conclusion. We leave that verdict to the readers.

**Reviewer #3:**

I am happy to see that the authors took the time and care to add additional details, methods, and figures to this manuscript. It has greatly strengthened the paper. I particularly like the addition of Fig 2. In its revised form, this manuscript is an important contribution to the study of seasonality in health and disease. Below I have included additional comments to be addressed before publication.

Thank you.

Major comments.

Fig 1A. I am confused by the first panel that has the trend fit between the healthy and unhealthy population. More description is needed to interpret this plot.

Figure 1A’s first panel describes the scenario in which two populations – a healthy one (the blue line) and an unhealthy one (the red line) – enrolled in the data at different times. The blue and red lines represent the trend of the diagnosis rate (DR) for the two populations. We can see that the healthy population joined and left our data earlier than the unhealthy one. Thus, if we fit a simple linear regression model, the result may lead us to conclude there is an upward trend of diagnosis rate. Nonetheless, the real trend is actually constant if we had the ability to collect the lifetime data of all patients (synchronous enrollment) for both populations. The trend of the linear fit (the orange line) comes from “asynchronous enrollments” of populations in various health statuses. We added this explanation to the figure legend.

Please provide information about the data structure for the data sets on gitbub. Currently there is no metadata and the files are not usable outside of the python code. In addition to your github, it would be better to put the manuscript data and code in a dedicated data archive, such as Dryad where it will have a DOI and appropriate metadata.

We have now provided a comprehensive description of the data structure in the README file on the project web site (<https://github.com/hanxinzhang/seasonality>). We stored the data of each condition (disease observations for a particular age-sex group) in a hierarchical python dictionary. The .bpkl3 files in the data directory are python3 pickle files serialized in the binary mode. Unpickling the file will give us a hierarchical dictionary of the following structure:

```
- cond
    - Condition                               # condition name
    - Data                                    # diagnosis data
        - (In-time, Out-time, Sex, Age-range) # 600 population strata enrolled in
                                              # different periods
            - HolidaySmoothObservation        # Smoothed diagnosis counts at different times
            - RealObservation                 # Diagnosis counts at different times
            - PopSize                         # Population size at different times
```

Again, more information about the data and sample code about how to use it can be found on the project web site (<https://github.com/hanxinzhang/seasonality>).

We also deposited data in the Dryad repository. The DOI has been assigned and the data will be made publicly available once Dryad staff completes their quality control checks. <https://doi.org/10.5061/dryad.vdncjsxv6>.

The plots of how well the models fit the raw data should be included in the supplement and not just github.

We have now included all the result figures, including the posterior fit plots, in the new revision, in a zipped, auxiliary file.

Minor comments.

line 81. "millions" should be million

We corrected the misspelling.

Fig 1B. The grey lines for the linear fits are very distracting/overwhelming in the plot. Perhaps make the thick grey lines a bit thinner or make them all more transparent.

We made the lines thinner and more transparent in the new version.

Fig 2. Please define what "In: and Out:" indicate for each stratum, I am assuming it is enrollment and disenrollment date. Also, it is really hard to distinguish the green from the blue time series, perhaps change the green to yellow or another color.

*In*: indicates the time the stratum joined in our data (enrollment beginning), and *Out:* indicates the time they left (enrollment end). We have made this clearer by adding an explanation in the new figure legend. We have recolored the green lines to orange to make them more distinguishable.

line 175. "out of the ordinary" seems to be subjective/odd phrasing because it is not clear what is the "ordinary" pattern.

We changed the expression to “distinctive seasonality,” which better reflects our intended meaning.

lines 323. Saying that your corrected analysis has "nonsensical results" seems subjective and to belittle all of your corrected results. I recommend rephrasing this. You can just say that some of the corrected results are not in line with previously published studies; correcting by all visits may not be optimal because seasonal variation in all visits may not be a good proxy for health seeking behavior, but more of a proxy of the seasonality of particular dominant ailments.

We rephrased the paragraph and avoided strongly-worded sentences. In the new revision, we stated:

“Third, our attempt to account for the seasonality of individual diseases rates by adjusting for the total rate of healthcare-seeking events (see Figs 6 and 7) produced results inconsistent with the findings of previous studies. … The results suggest correcting by all visits may not be optimal because the seasonality of all visits does not reflect non-specific, health-seeking behaviors. The evidence shows this is more likely determined by the seasonality of the dominant disease.”

line 361. You should not use "her" when referring to SAD patients, it indicates they are all female.

Changed to “their.”

line 390 paragraph. It might be helpful to also discuss here that healthcare coverage differs for these countries and may impact health seeking behavior as well and the diagnosis rate. Without nationalized health care, people with chronic diseases (such as psychiatric disorders) who lack health insurance may be less likely to seek treatment unless it is an emergency or they are having an acute episode.

We added the following reasoning:

“Also, healthcare coverage and country-specific policies may explain curve differences. Without a universal health care system, such as the one in Sweden, people with chronic psychiatric diseases who lack comprehensive health insurance plans may be reluctant to seek medical care until they experience an acute emergency.”

Discussion. It would be worth mentioning in the discussion that in the US there are school-based mental health awareness programs. Thus, some of the summertime dip in depression/anxiety diagnosis for 5-18 year-olds could be due in part to underreporting when students are out of school. I don't know much about these programs, but did a quick google scholar search to try and find info. Here is one paper: [https://urldefense.com/v3/__https://www.ncbi.nlm.nih.gov/pmc/articles/PMC5123790/__;!!OaZRxjSJGsACmqMyc8s!dnbPnEOji-B9jZQisBptrj4Nm-DsY8-fOCLbUh7TgZmUXCN4-xOJ_2DVl6uQ6JuuWEnFEOiBS1zpmA$](https://urldefense.com/v3/__https:/www.ncbi.nlm.nih.gov/pmc/articles/PMC5123790/__;!!OaZRxjSJGsACmqMyc8s!dnbPnEOji-B9jZQisBptrj4Nm-DsY8-fOCLbUh7TgZmUXCN4-xOJ_2DVl6uQ6JuuWEnFEOiBS1zpmA$)

We added a new paragraph to discuss the possible connection between the increase in psychiatric diagnoses during school time and certain social patterns (such as summer vacation and mental health awareness programs):

“Besides, the observed seasonality in some psychiatric diseases may be rooted in certain culture-specific events. For example, in the summer, the decrease in ADHD visits in school-aged adolescent (Figs 3 and 6) may be associated with summer vacation travels, affecting both physicians and patients. Additionally, mental health awareness programs in some US K-12 schools may also increase psychiatric diagnoses during school time (spring and autumn).”
